# Offline stimulation of human parietal cortex differently affects resting EEG microstates

**DOI:** 10.1038/s41598-018-19698-z

**Published:** 2018-01-19

**Authors:** Pierpaolo Croce, Filippo Zappasodi, Paolo Capotosto

**Affiliations:** 1 Department of Neuroscience Imaging and Clinical Science, “G. d’Annunzio” University, Chieti, Italy; 2 Institute for Advanced Biomedical Technologies, “G. d’Annunzio” University, Chieti, Italy

## Abstract

The interference effects of transcranial magnetic stimulation (TMS) on several electroencephalographic (EEG) measures in both temporal and frequency domains have been reported. We tested the hypothesis whether the offline external inhibitory interference, although focal, could result in a global reorganization of the functional brain state, as assessed by EEG microstates. In 16 healthy subjects, we inhibited five parietal areas and used a pseudo stimulation (Sham) at rest. The EEG microstates were extracted before and after each stimulation. The canonical A, B, C and D templates were found before and after all stimulation conditions. The Sham, as well as the stimulation of a ventral site did not modify any resting EEG microstates’ topography. On the contrary, interfering with parietal key-nodes of both dorsal attention (DAN) and default mode networks (DMN), we observed that the microstate C clearly changes, whereas the other three topographies are not affected. These results provide the first causal evidence of a microstates modification following magnetic interference. Since the microstate C has been associated to the activity in regions belonging to the cingulo-opercular network (CON), the regional specificity of such inhibition seems to support the theory of a link between CON and both DAN and DMN at rest.

## Introduction

Repetitive Transcranial Magnetic Stimulation (rTMS) provides a unique opportunity of studying relations between brain activity and behaviour in healthy humans. Importantly, the rTMS interferes not only with the local activity of the stimulated site, but also with the activities of anatomically and functionally connected areas^1^. When combined with other neuroimaging techniques, rTMS effects can be used both to establish a causal link between brain activity and task performance, and to explore the functional brain connectivity^2–4^. In this context, in recent years, a series of studies was performed by combining rTMS and Electroencephalography (EEG) or Magnetoencephalography (MEG) to investigate the effect of online/offline magnetic stimulations on electrical brain activity. In particular, online rTMS over specific brain regions during the execution of cognitive tasks interferes with EEG potentials^2,3^ as well as with EEG/MEG rhythms^2,4–6^ and performance^7,8^. Furthermore, offline inhibitory interference with spontaneous ongoing- i.e. not task-driven- activity has been reported. Indeed, rTMS over parietal regions specifically modulated the alpha rhythms observed in resting state EEG^9^.

An alternative way to globally represent the temporary brain activity resulting from concomitant active networks is the microstate analysis^10^. Indeed EEG microstate topographies have been shown to be stable for periods of about 40–120 ms^10^, whereas the strength of the electric field, quantified by computing the standard deviation across channels measurement at each time point (Global Field Power, GFP), may change. Through a variety of clustering algorithms^11^, it is possible to represent the EEG time course as sequences of different microstates. Such a topographical approach does not require any type of a priori hypothesis and can give a more informative framework and global interpretability^11^ than other EEG analysis techniques, which aim at evaluating the brain’s potential by a priori choice of electrodes of interest, at determinate time intervals or in specific frequency bands^12,13^. Four typical topographies, explaining about 80% of the EEG variance, have been obtained in healthy adults^14^. These templates have been labeled as A, B, C and D: A and B had a nearly vertical orientation (respectively from left occipital-parietal to right fronto-central for A and the opposite for B), while C and D had a horizontal orientation (a symmetrical back to prefrontal orientation for C and a central positivity with an occipital to fronto-central symmetrical orientation for D). Microstate analysis has been proved to be useful to reveal the significance of modular aspects of brain dynamics and their role in behavioural control, as well as for the characterization of brain diseases^12,13,15–18^. In a recent study^17^, reported a correlation between the BOLD activations in regions belonging to different human resting state brain networks and the four microstate topographies. Specifically, they were associated to phonological (A), visual (B), cingulo-opercular (C) and attention reorienting (D) systems^17^. In our knowledge, to date, no previous studies investigated the effect of magnetic stimulation over EEG microstates. Since a microstate may be associated to a functional brain state during the occurrence of specific neural processes, it can be hypothesized that the inhibition of the activity of a brain area, although focal, could result in a global reorganization of the functional brain state and thus in different patterns and timing of microstate topographies and/or metrics, respectively.

**Figure 1:**
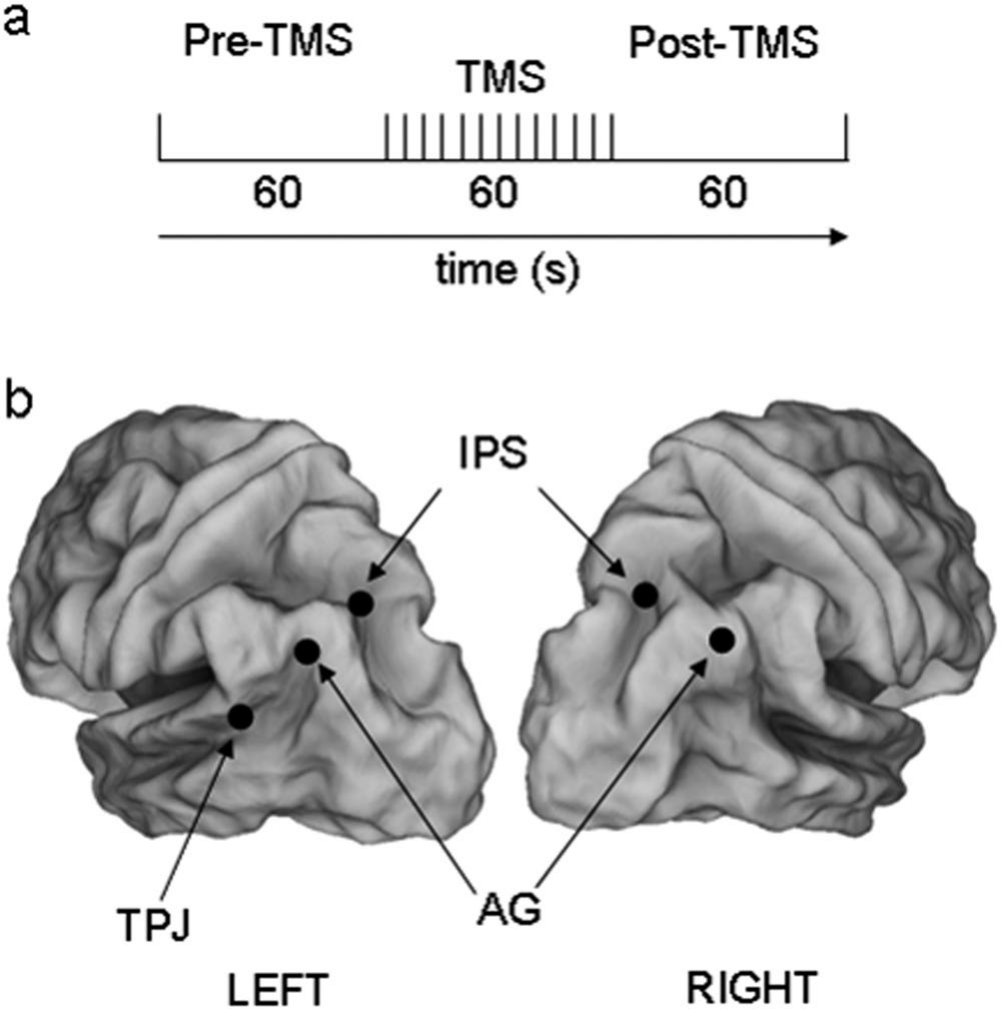
Experimental design: (**a**) Temporal sequence for each rTMS stimulation condition. (**b**) View of the left and the right hemispheres atlas brain with regions obtained from a meta-analysis studies^3,9^. Regions stimulated with rTMS in this experiment correspond to the following MNI coordinates (x, y, z in mm): right IPS: 23, −65, 48, right AG: 53, −67, 46, left IPS: −25, −63, 47, left AG: −47, −67, 36, and left TPJ: −52, −49, 17.

To verify this hypothesis, we explored the possible effects of the offline external inhibitory interference with the EEG microstates, by combining rTMS with EEG recordings at rest. EEG microstates before and after the stimulation of several brain regions were extracted. Specifically, we interfered with the neural activity of different parietal sites (Fig. 1): two key-nodes of the Dorsal Attention Network (DAN), i.e. the left and the right Intra-Parietal Sulcus (pIPS); two crucial areas of the Default Mode Network (DMN); i.e. the right and left Angular Gyrus (AG); a more ventral parietal region that does not belong to both DAN and DMN, i.e. the left Temporo-Parietal Junction (TPJ). We compared the four typical resting EEG microstates’ topography and metrics in the period that precedes and follows magnetic inhibition within and between each active and non-active (Sham) TMS conditions.

## Results

In 12 subjects, out of 16, the optimal number of microstates were found to be equal to 4 in all conditions, obtained by applying the CV and KL criteria. The optimal number was 3 before and after pIPS, AG and TPJ stimulation in 3 subjects and was 5 in all conditions in only 1 subject. Thus, the optimal number of microstates was chosen equal to 4. The Global Explained Variance was not different across conditions, as shown by the lack of significant effects or interactions in ANOVA design (consistently p > 0.150; mean and standard deviation across conditions: 65.9 ± 5.6%).

**Figure 2:**
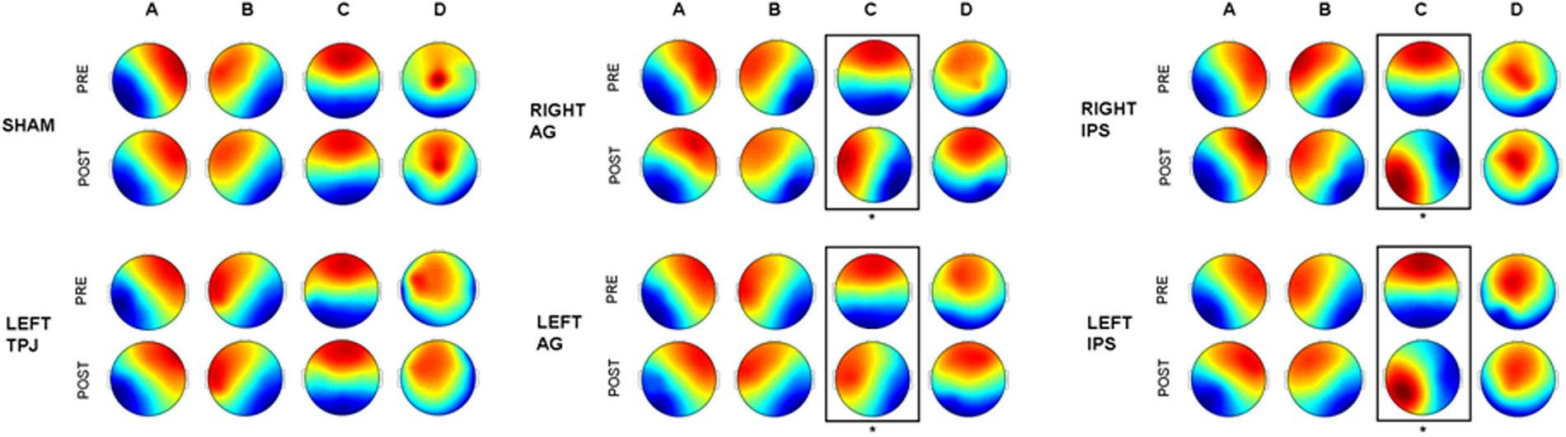
Topography of the four common resting EEG microstates in each rTMS condition (i.e. Sham, left TPJ, left AG, right AG, left IPS, right IPS) in the period that precedes (1 min, pre-TMS) and follows (1 min, post-TMS) the magnetic stimulation. Post-hoc test (performed by TANOVA): statistically significant differences between pre- and post-TMS periods in each condition are indicated by one asterisk (p < 0.005).

Figure 2 illustrates the four EEG microstates’ topography separately in the two periods of interest, before and after stimulation, for the six TMS conditions. The canonical 4 templates A, B, C and D, previously found in the literature, can be observed in all conditions, except after AG and IPS stimulation. By comparing the microstates’ topographies, the first analysis aimed to observe whether the four common resting EEG microstates’ topographies differ in the baseline period across the different stimulation sites, i.e. active (left and right IPS, left and right Ag, left TPJ) and non-active (Sham). To this aim, TANOVA was applied to the 6 conditions pre-rTMS. No differences were found (p > 0.500). Successively, we compared the microstates’ topography before and after stimulation, separately for each stimulated site by TANOVA. After the Sham stimulation, we did not detect any significant modification (p > 0.500). Similarly, when magnetic stimulation was delivered over the TPJ no statistically significant difference (p > 0.200) was observed between the microstates’ topographies in the two periods of interest. On the other hand, we observed significant differences by comparing the pre- and post- TMS periods within each DAN and DMN regions (Fig. 2). Post hoc tests, performed by dissimilarity index, indicated that magnetic stimulation over both left (p < 0.001) and right (p < 0.001) AG, as well as over both left (p = 0.003) and right (p < 0.001) IPS, significantly changes the resting state EEG microstate “C” topography. Notably, no differences (p > 0.2) were reported in the other three microstates after stimulation of all DAN and DMN regions.

When applying the ANOVA design to microstate metrics, a significant Microstate X Site interaction was found [duration: F (5.4, 81.1) = 15.192; p < 0.001; occurrence: F (4.9, 73.4) = 18.377; p < 0.001; coverage: F (5.8, 86.6) = 20.716; p < 0.001]. This interaction indicates that the microstate metrics differently changed after rTMS according to the stimulated site (Table 1). In particular, post-hoc t-test analysis (Bonferroni corrected) indicated that after the non-active Sham condition a decrease of microstate A and an increase of microstate B metrics with respect to the baseline values were observed (Table 1). After AG stimulation in the left hemisphere a decrease of microstate D metrics was found. On the contrary, microstate D metrics increased after right AG stimulation and both A and B values increased. After the stimulation of both left and right IPS, not only the topography of microstate C changed, but also this “new” microstate presented higher metrics. Moreover, only after stimulation of right IPS a decrease of microstate D and an increase of microstate A metrics were found. When stimulating the TPJ, no effects were found.

**Table 1:** Microstate metrics (mean and standard deviation) in the pre-TMS, post-TMS and difference between pre-and post- stimulation. Stars indicate significant differences, as assessed by two-tailed paired t-test (Bonferroni corrected).

	Pre TMS				Post TMS				Post vs Pre rTMS difference			
	A	B	C	D	A	B	C	D	A	B	C	D
**DURATION (ms)**												
Left AG	38.7	42.7	40.9	33.3	41.9	41.7	45.5	25.8	3.2	−1.0	4.6	−7.5*
	3.7	5.0	4.3	1.4	3.2	2.4	8.4	5.5	1.2	1.5	2.6	***1.2***
Right AG	43.2	41.7	37.9	31.8	35.9	37.4	41.3	40.0	−7.3*	−4.3*	3.4	8.2*
	4.3	4.5	2.8	3.3	2.4	2.8	2.9	2.5	***1.0***	***1.1***	1.1	***1.1***
Left IPS	39.8	39.7	37.8	37.8	40.2	37.2	43.6	35.4	0.4	−2.5	5.8*	−2.4
	3.4	2.4	1.9	3.5	2.7	3.5	4.5	3.4	1.2	0.8	***1.2***	1.4
Right IPS	36.1	39.6	37.1	42.9	40.2	37.8	44.5	31.0	4.1*	−1.8	7.4*	−11.9*
	2.6	2.2	2.2	3.6	2.6	3.2	4.8	3.9	***1.0***	0.6	***1.3***	***1.7***
TPJ	41.4	41.0	43.4	34.2	41.0	40.9	43.9	35.3	−0.4	−0.1	0.5	1.1
	2.6	3.1	5.3	6.7	2.7	4.3	3.1	2.8	1.0	1.0	1.5	1.7
Sham	42.7	40.7	39.2	32.7	40.6	44.2	39.4	32.1	−2.1	3.5*	0.2	−0.6
	3.5	3.6	3.4	4.6	3.4	3.8	4.3	3.9	0.7	***0.7***	0.9	0.6
**COVERAGE (%)**												
Left AG	24.5	31.1	27.8	16.6	29.7	29.7	32.4	8.3	5.2	−1.4	4.6	−8.3*
	4.5	5.0	5.8	2.9	3.4	4.6	6.7	8.0	1.7	2.2	2.8	***1.5***
Right AG	32.0	29.2	24.2	14.6	20.3	22.7	29.5	27.5	−11.7*	−6.5*	5.3	12.9*
	5.7	5.1	4.2	4.4	3.4	3.7	3.5	3.9	***1.6***	***1.4***	1.7	***1.6***
Left IPS	26.5	26.4	23.2	23.9	27.0	22.2	31.2	19.6	0.5	−4.2*	8.0*	−4.3
	3.3	2.5	2.9	3.6	3.6	4.6	5.2	5.2	1.4	***1.0***	***1.8***	1.7
Right IPS	21.2	26.3	22.2	30.3	28.4	24.1	33.6	13.9	7.2*	−2.2*	11.4*	−16.4*
	4.1	2.8	2.7	4.8	3.8	3.6	4.3	5.1	***1.7***	***0.4***	***1.6***	***2.1***
TPJ	27.7	27.4	29.1	15.9	26.6	26.3	29.4	17.8	−1.1	−1.1	0.3	1.9
	3.4	3.9	5.9	8.3	3.3	5.3	3.2	4.7	1.4	1.4	1.6	2.1
Sham	31.1	27.7	25.6	15.6	26.9	32.6	24.9	15.5	−4,2*	4,9*	−0,7	−0,1
	4.3	4.3	4.0	7.9	4.4	4.0	4.6	6.3	***0,9***	***0,7***	1,0	0,9
**OCCURRENCE (microstate per second)**												
Left AG	6.3	7.3	6.8	4.9	7.1	7.1	7.2	2.9	0,8	−0,2	0,4	−2,0*
	0.6	0.4	0.8	0.7	0.5	0.8	0.6	2.4	0,3	0,3	0,3	***0,5***
Right AG	7.4	7.0	6.3	4.4	5.5	6.0	7.1	6.9	−1,9*	−1,0*	0,8	2,5*
	0.7	0.6	0.8	1.0	0.8	0.7	0.6	0.6	***0,2***	***0,2***	0,3	***0,3***
Left IPS	6.6	6.6	6.1	6.3	6.7	5.8	7.1	5.4	0,1	−0,8*	1,0*	−0,9
	0.5	0.4	0.6	0.6	0.5	0.8	0.6	1.0	0,2	***0,2***	***0,3***	0,3
Right IPS	5.8	6.7	6.0	7.0	7.1	6.4	7.7	4.3	1,3*	−0,3	1,7*	−2,7*
	0.8	0.5	0.7	0.7	0.7	0.6	0.4	1.2	***0,3***	0,1	***0,2***	***0,4***
TPJ	6.7	6.7	6.6	4.2	6.5	6.4	6.7	5.0	−0,2	−0,3	0,1	0,8
	0.5	0.6	0.9	1.4	0.5	0.7	0.4	1.0	0,2	0,2	0,2	0,3
Sham	7.2	6.8	6.5	4.8	6.6	7.4	6.3	4.6	−0,6*	0,6*	−0,2	−0.2
	0.6	0.6	0.5	1.9	0.7	0.4	0.6	1.4	0,1	0,1	0,1	1,3

## Discussion

We used a novel approach in which EEG microstates are evaluated before and after the magnetic stimulation over specific parietal regions. Our results showed that the focal inhibition of specific areas results in a modification of topography patterns of EEG microstates, as well as in a variation of their dynamics. In particular, when bilateral IPS and AG, but not TPJ, are inactivated, the topography of the EEG microstates C changes, whereas no differences were observed in the other three common EEG microstates’ topographies. In the same way, also the metrics of microstate C changed dependent on the stimulated site. In particular, they increased after bilateral IPS and AG stimulation, but not after TPJ. Importantly, no changes in topography or metrics of microstate C, as well as in the other 3 common microstates topographies, were observed after Sham stimulation.

As the topographies of microstates originate from the synchronous activities of neuronal assemblies reflecting different functions^15^, this new pattern of microstate C expresses a global re-organization of brain activity of different brain areas. On the other hand, an increase of the microstate metrics can be interpreted as stability or engagement of the neural activity of the network generating the microstate topography and conversely a decrease may be a sign of an hypoactivity.

Previous works showed that a modification of microstate topography and metrics is possible by cognitive manipulation^19^. Moreover, altered topographies and metrics have been found in the acute phase of the stroke as a consequence of a mono-hemispheric lesion^18^, as well as in neurodegenerative or psychiatric diseases^20^. Our study adds the evidence that the topography and metrics of microstates can be modified also by active inhibition by means of magnetic stimulation.

A combined EEG-fMRI study associated the microstate C to the cingulo-opercular network (CON^17^,). Even if this study reported eyes closed microstates, we may extend this association also for our eyes open topographies to hypothesize possible causal interactions between networks. Indeed, the 4 canonical microstate topographies were found both in the eyes closed and eyes open condition^19,21,22^, although it has been argued that more than 4 canonical microstates should be considered in eyes open condition to explain a similar amount of variance of a eyes closed condition^19^.

The CON has been associated with preservation of tonic alertness providing stable task ‘set-maintenance’^23–26^ and meets the general criteria for a domain-general self-regulation system. Nevertheless, its function is not easy to characterize due to its pervasive activity and the co-activation with other brain networks. In particular, the possible interaction between the CON and both DAN and DMN is still not directly disclosed. In this regard, several neuroimaging studies showed that regions of the DAN and DMN exhibit an antagonistic (push-pull) pattern of response during attention and memory, respectively^27–29^. Such push-pull pattern is consistent with the pattern of negative correlation of BOLD signal observed at rest^30^. This dynamic functional competition is in line with behavioral evidence indicating that internally-oriented attention impairs externally directed tasks^31,32^ and, vice versa, that mind-wandering decreases as a function of the difficulty of the externally-oriented task (reviewed in^33^). These reciprocal interactions suggest either a cross-inhibitory mechanism, such that increases in one network directly leads to decreases in the other, or an indirect mediation by a third-party source (CON) that dynamically links with each of the two networks as function of task demands. According to a direct cross-inhibition hypothesis, one should predict that the inactivation of a region belonging to the DMN during attention task, as well as the inactivation of a key- node of the DAN during memory task, has a positive influence on visual performance. Instead, combining EEG recording with repetitive TMS, we recently reported no interaction between suppression of task irrelevant sites and behavioral performance as compared to a non-active TMS condition^5^. Hence, such causal evidence indirectly supports the hypothesis that DAN-DMN interaction is indirectly mediated by a higher-order prefrontal network involved in the task-set maintenance (CON), consistently with previous neuroimaging studies showing a dynamic task-dependent functional interaction between DAN/DMN and the CON^23,27^). Nevertheless, the two above hypotheses came only from correlative findings since no previous studies causally investigated the relationship across these human brain networks. More in general, it can be assumed the presence of a direct interaction between two networks when inhibition of regions belonging to one network causes effects on the activity of the other one. Here, we report that whereas TMS over bilateral IPS and AG similarly modified the organization of the microstate “C”, interference over left TPJ did not change any of the resting EEG microstates. At the present early stage of the research we can only speculate on these results. Nevertheless, since the topography of the resting EEG microstate previously associated to the CON was clearly modified only after inactivation of crucial nodes of both DAN and DMN, our results seem to support the hypothesis of an indirect mediation of the two networks (DAN and DMN) by a third-party source (CON), thus supporting the CO network characterization as a control system and providing novel inputs for the current understanding of the organization of the human brain networks.

In addition to the increase of microstate C metrics, we observed differences also in the metrics of the other three EEG microstates (A, B and D), which selectively depend on the stimulation site. Seitzman *et al*.^34^ suggested that the microstate metrics could depend on the state of the visual system, related to the amount of alpha activity. In particular, comparing the eyes closed with the eyes open condition, a decrease of microstate A together with an increase of microstate B have been observed^34^. We found the same trend of metrics of microstates A and B after the Sham stimulation. This behavior could be hypothesized to be related to a progressive wandering of attention and decline in arousal during the EEG recording, independently from the stimulation. Indeed, an alpha power increment in the rest period across time has been previously described and related to progressive disengagement of cortical areas^35–37^. The absence, or even the inversion, of this modulation in the other conditions compared to Sham can be explained by the effect of the real stimulation on specific sites. Moreover, Miltz *et al*.^38^ recently demonstrated that the four microstate topographies are differently determined by spatial distribution and strength of intra-cortical alpha oscillations. The microstate C, compared to the other microstates, shows the stronger alpha activity in wide spread cortical regions beyond the ACC, including the left and right posterior areas, sign of a large-scale process involving both posterior and frontal areas. Conversely, the microstates A and B showed an interhemispheric difference of alpha prevalence, i.e. strong alpha in left posterior region for microstate A and in right posterior region for microstate B^38^.

We documented that the selective inactivation by rTMS of right IPS corresponds to lower metrics of microstate D. An association between right IPS and microstate D has been already described. In fact, the microstate D has been inversely correlated with the BOLD signal in right dorsal lateral and ventral areas of the frontal and parietal cortex^17^, brain areas identified as belonging to the DAN^39^. As pointed out by^17^ a negative correlation between microstate time course and BOLD signal does not automatically imply neuronal de-activation. Indeed, this association was confirmed by^34^, in which the microstate D metrics increased during a task involving the DAN.

Finally, an opposite behavior was found for microstate D metrics after the stimulation of AG in the two hemispheres: an increase after right AG and a decrease after left AG stimulation. AG is one of the major connecting hubs at the system level and its role can clearly be understood only in parallel with the interaction and influence from other regions^40^. Opposite behavior following the AG activity interruption of left and right hemisphere has been already observed for both neurophysiological activity and functional abilities. Indeed, a right lateralization of alpha activity coherence has been described after rTMS of AG^5^. Moreover, while a damage of left AG is paired to decrement of word processing, the electrical stimulation of right AG triggers out-of-body experience and is paired to alteration of visual spatial attention^40^.

As a final methodological limitation of this work, we note that the present microstate analysis was performed in broadband frequency and not in frequency-dependent bands. This latter analysis might provide the impact of the TMS on the link between EEG microstates and brain rhythms. However, to date, a clear relationship between EEG microstate’s topography and EEG rhythms is still not disclosed^41^. Thus, we used a more consolidated analysis (i.e. in broadband frequency) aiming to test whether the offline focal stimulation might result in a global reorganization of the functional brain state, as assessed by the well reported typical four EEG microstates. Nevertheless, after a clear identification of the link between EEG microstates and brain rhythms, future dedicated studies should deeply address the effect of magnetic stimulation over specific brain regions at different frequencies.

In conclusion, our data demonstrated that the selective inhibition by rTMS of one cerebral area re-organizes the global brain activity as described by EEG microstates. This method can be useful to better understand the organization of the human brain networks and to infer causal relationships between the activity of different brain networks.

## Materials and Methods

### Subjects and stimuli

Sixteen healthy adult volunteers (age range: 21–27 yrs old; 6 females) with no previous psychiatric or neurological history participated to the experiment. All subjects were right-handed, as assessed by the Edinburgh Inventory test and their vision was normal or corrected-to-normal. All experiments were conducted with the understanding and written informed consent of each participant, according to the Code of Ethics of the World Medical Association, and the standards established by the University of Chieti Institutional Review Board and Ethics Committee. The experimental protocol was approved by the Ethics Committee of “G. d’Annunzio” University of Chieti-Pescara. Some results from this dataset have been previously published^9^. Subjects were seated in a comfortable reclining armchair. They maintained fixation on a small white cross stimulus (subtending 0.7° of visual angle) displayed on a black background in the centre of a computer screen positioned at a distance of 80 centimetres.

### Procedures for rTMS and identification of target scalp regions

Repetitive TMS, used to interfere with neural activity, was delivered through a focal, figure eight coil (outer diameter of each wing 7 cm) connected with a standard Mag-Stim Rapid 2 stimulator (maximum output 2.2 Tesla). The rTMS train was delivered based on the following parameters: 1-minute duration, 1-Hz frequency and intensity set at 100% of the individual motor threshold. These parameters are consistent with published safety guidelines for TMS stimulation^42^. Individual resting excitability threshold for right motor cortex stimulation was preliminarily determined following standardized procedure^43^. Of note, 1-Hz rTMS for 1 minute has been proved to inhibit the target cortical area for 1 or 2 minutes post-stimulation^9^. The experimental design included six conditions, applied in different blocks, and randomized across subjects. Each subject performed all the conditions. Two consecutive TMS sessions were separated by an interval of about 10 minutes. In the “Sham” condition, a pseudo rTMS was delivered at scalp vertex; stimulation was ineffective due to the reversed position of the coil with respect to the scalp surface (i.e. the magnetic flux was dispersed to air). In the five active conditions the centre of the coil wings was located on the scalp at a position corresponding to different cortical regions obtained from meta-analysis studies^44^ (Fig. 1b). Two of these regions corresponded to core regions of the DAN, i.e. the right and the left Intra-Parietal Sulcus (pIPS; MNI coordinates: 23, −65, 48 mm and −25, −63, 47 mm respectively). Two other regions are hubs of the DMN: the right and left Angular Gyrus (AG; x, y, z: 53, −67, 46 mm and −47, −67, 36 mm). The last region was used as active control since it does not belong to both networks and takes place in the left temporal-parietal cortex: left Temporo-Parietal Junction (TPJ; x, y, z: −52, −49, 17 mm). The location of the stimulation sites were automatically identified on the subject’s scalp using the SofTaxic navigator system (E.M.S. Italy, www.emsmedical.net), which uses a set of digitized skull landmarks (nasion, inion, and two pre-auricular points), about 40 scalp points entered with a Fastrak Polhemus digitizer system (Polhemus) and an averaged stereotaxic MRI atlas brain in Talairach space. The average Talairach coordinates in the SofTaxic navigator system were transformed through a linear transformation to each individual subject’s scalp. Such method has an error of about 5 mm over a method in which each subject’s own MRI is used for localization^45^ and has been proven to be successful across different stimulation parameters^9,46^. Of note, in the present study none of the subjects declared any kind of discomfort or pain during each experimental conditions and after the experimental sessions.

### Electroencephalography recordings

EEG data were recorded (BrainAmp; bandpass, 0.05–100 Hz, sampling rate, 256 Hz; AC couple mode recording) from 32 EEG electrodes placed according to 10–20 augmented system, and mounted on an elastic cap resistant to magnetic pulses. Electrode impedance was below 5 kΩ. Two electro-oculographic channels were used to monitor eye movement and blinking. The acquisition time for all conditions was set from −1 to +0 min before rTMS train onset, and from +1 to +2 min after the rTMS train onset. EEG data were segmented off-line in windows of 2 sec. Notably, the EEG single trials contaminated by eye movement, blinking, or involuntary motor acts (e.g. mouth, head, trunk or arm movements) were rejected off-line. The EEG data analysis was performed in the following periods of interest: (i) “pre-TMS” (1 minute before rTMS train), (ii) “post-TMS” (1 minute after rTMS train) (Fig. 1a).

### Microstates extraction

EEG data were filtered between 1 and 40 Hz (Butterworth filter of 2_nd_ order, forward and back filtering). Periods of one minute of EEG recording before and one minute after the end of magnetic stimulation were considered. These two epochs were segmented in windows of 2 seconds of duration for microstate extraction. A modified version of the k-means clustering algorithm^19^ was applied. Firstly, the Global Field Power (GFP) was calculated for each time frame as the standard deviation of EEG signal across electrodes. Only the EEG data corresponding to the GFP maxima were then submitted to the clustering algorithm^11^. For each subject, for each condition (pre and post rTMS) and for each stimulated site (left left and right AG, left and right IPS, TPJ, Sham), the k-means algorithm was repeated to find a number of topographic templates between 1 and 10. Since this algorithm requires the number of clusters to be specified, the optima number was chosen by calculating, for each number of template, both the cross validation (CV) and the Krzanowski-Lai (KL) criteria and by choosing the number of clusters which corresponded to the second maximum value of the KL and the minimum value of the CV^11^. Hence, subject wise templates were extracted from the original data. Then, conditions-wise mean templates were computed re-clustering the maps extracted for each subject and for each of the 12 conditions in the following way. Separately for each condition, four random maps were selected from the subject-wise maps as initial templates. Then the four maps of each subject were assigned to each template considering the best fit of the correlation between them. The new templates were obtained by following the procedure in^16^. This procedure was repeated 30 times choosing the iteration with the lowest inter-subject variance. The four templates resulting from this procedure were paired between groups, based on the minimum value of maps global dissimilarity (see^16^ for details). The global dissimilarity between two maps ${\rm{u}}$ and ${\rm{v}}$ is defined as^11^:

1 $${D}_{u,v}=\sqrt{\frac{1}{N}\sum _{i=1}^{N}{(\frac{{u}_{i}}{GF{P}_{u}}-\frac{{v}_{i}}{{\rm{G}}F{P}_{v}})}^{2}}$$

where, *u*_i_ and *v*_i_ are the electric potentials of the *i*_th_ electrode for the maps *u* and *v*, respectively, *GFP*_u,v_ are the global field powers of the maps and N is the number of electrodes^47^.

The conditions-wise templates were then fitted backward to the original data to compute the metrics of the microstates. The back-fitting procedure considers the maximum correlation between each template and the topography at each time instant. Then for each subject, for each microstate class of each conditions, the following metrics were calculated^48^:

1. Mean microstate duration: average time covered by a single microstate class.
2. Mean percentage of covered analysis time: percentage of time covered by a single microstate class.
3. Mean occurrences per second: mean number of distinct microstates of a given class occurring within a 1 second window.

Complete microstate analysis was performed with the free available Cartool Software^48^.

### Statistical analysis

To test the differences between topographies within pre- and post- TMS periods and among the different stimulated sites, Topographic ANalysis of VAriance (TANOVA, see^47^ for details) was performed. Statistical significance was assessed with a p-value of TANOVA was lower than 0.05. Subsequently, only for those templates resulting above the statistical significance from the TANOVA, post-hoc tests were performed to assess the differences between 2 specific groups. The post-hoc analysis was performed by a non-parametric test based on the global dissimilarity^47^.

To assess difference in post stimulation metrics with respect to pre stimulation values, separately for duration, coverage and occurrence a repeated measure Analysis of Variance (ANOVA) was performed on the post vs pre differences, with Microstate (A, B, C and D) and Site (left AG, right AG, left IPS, right IPS, TPJ, Sham) as within-subject factor. Greenhouse-Geisser correction has been applied when the sphericity assumption was not valid. Post-hoc comparisons were performed to assess significant difference of post vs pre values. Bonferroni correction was applied.
